# Airway Necrosis and Fungal Plaque Causing Central Airway Obstruction Due to Candida auris: A Case Report

**DOI:** 10.7759/cureus.93075

**Published:** 2025-09-23

**Authors:** Sofia Pouriki, Theoni Agapitou, Anastasia Kosmidou, Asimina Rautopoulou, Vasiliki Nanou, Panagiota Manthou, Ioannis Nikolopoulos, Zafiria Mastora

**Affiliations:** 1 Intensive Care Unit, General Hospital of Thoracic Diseases "Sotiria", Athens, GRC; 2 Infection Control Office, General Hospital of Thoracic Diseases "Sotiria", Athens, GRC

**Keywords:** airway obstructive infections, candida auris, icu, nosocomial infections, severe acute respiratory

## Abstract

*Candida auris* is an emerging multidrug-resistant fungal pathogen associated with high morbidity and mortality, particularly in critically ill and immunocompromised patients. To our knowledge, airway-obstructing tracheobronchial disease due to *C. auris* has not been previously reported.

An 80-year-old woman with multiple comorbidities, including myelofibrosis under erythropoietin therapy, presented with severe coronavirus disease 2019 (COVID-19) pneumonia. On admission, clinical findings included fever, cough, dyspnea, leukocytosis (20.3 K/µL), elevated C-reactive protein (23.9 mg/dL), and procalcitonin (2.12 ng/mL). Chest CT revealed diffuse consolidations and ground-glass opacities. Progressive hypoxemia necessitated intubation and mechanical ventilation. The clinical course was complicated by ventilator-associated pneumonia caused by multidrug-resistant *Acinetobacter baumannii*, treated according to antibiogram results. Severe neuromuscular weakness prevented extubation, leading to tracheostomy. Shortly thereafter, progressive respiratory failure developed. Bronchoscopy demonstrated extensive necrosis and narrowing of the trachea. Tissue biopsy and cultures confirmed *C. auris* infection. Histopathology demonstrated fungal spores and septate hyphae within necrotic tracheobronchial mucosa on periodic acid-Schiff/Grocott methenamine-silver (PAS/GMS) stains, consistent with tissue invasion rather than surface colonization. No angioinvasion was identified. The presence of organisms infiltrating the necrotic mucosal tissue, together with obstructing fungal plaques visualized bronchoscopically, supported invasive infection and excluded mere airway colonization. Antifungal therapy was administered, resulting in the complete resolution of tracheal necrosis and stenosis on follow-up bronchoscopy. Despite recovery from fungal infection, the patient subsequently developed septic shock secondary to *Klebsiella pneumoniae* bacteremia and died.

This case illustrates a rare presentation of *C. auris* tracheal infection leading to airway necrosis in an immunocompromised, mechanically ventilated patient. Early diagnosis through bronchoscopy, culture, and histopathology, combined with timely antifungal therapy, is critical for management. The case emphasizes the diagnostic and therapeutic challenges posed by multidrug-resistant fungal infections in critically ill patients with COVID-19.

## Introduction

Airway involvement due to *Candida auris* is exceptionally rare, with no established incidence data and only isolated case descriptions of tracheobronchial disease in the literature. In critically ill patients with coronavirus disease 2019 (COVID-19), immune dysregulation, characterized by lymphopenia, impaired innate immune responses, and the frequent use of corticosteroids or other immunomodulatory therapies, may further predispose to invasive fungal infections, potentially contributing to this unusual presentation. Central airway obstructive infections (CAOIs) are a rare complication caused by fungal, bacterial, parasitic, or viral pathogens. They often occur in different clinical syndromes with atypical anatomical distributions and poor outcomes [1]. Early recognition and rapid bronchoscopic treatments are required to relieve obstructive symptoms and prevent further respiratory compromise. In the intensive care unit (ICU) setting, the differential diagnosis for necrotizing central airway lesions includes mucormycosis and invasive aspergillosis, well-documented causes of fulminant tracheobronchitis in immunocompromised hosts [2,3], as well as noninfectious etiologies such as tracheal ischemia from prolonged or high-pressure endotracheal tube cuffs [4]. COVID-19-associated laryngotracheitis and necrotizing bacterial tracheitis have also been increasingly recognized during the pandemic era [5,6]. In the present case, these alternative etiologies were excluded based on negative cultures for molds and bacteria, absence of angioinvasion or hyphae typical of *Mucorales* or *Aspergillus* on histopathology, and lack of ischemic changes attributable to mechanical factors.

Recently identified as a multidrug-resistant pathogenic yeast, *C. auris* produces invasive infections and outbreaks that have a high death rate among hospitalized patients, especially those who have been admitted to ICUs [7-9].

Given the immunosuppressed state of many COVID-19 patients, close monitoring for fungal colonization and infection is imperative. Early identification and management of *C. auris* infections and complications are critical, as mortality rates in such cases have been reported to range from 30% to 72%, especially in bloodstream and abdominal infections [10,11].

We present a rare case of *C. auris* infection in a COVID-19-positive patient complicated by mycetoma in the trachea, resulting in severe airway obstruction.

## Case presentation

An 80-year-old woman with a recent positive severe acute respiratory syndrome coronavirus 2 (SARS-CoV-2) antigen test presented to the emergency department with weakness, dry cough, and shortness of breath for two days, along with fever up to 39°C over the preceding three days. Her past medical history included dyslipidemia, arterial hypertension, carotid stenosis, osteoporosis, and primary myelofibrosis, treated with erythropoietin. She was an ex-smoker with a 30-pack-year history and reported no drug allergies. The patient was considered immunocompromised due to underlying myelofibrosis with disease-related cytopenias, particularly anemia and relative lymphopenia, which are recognized risk factors for opportunistic infection; erythropoietin therapy itself is not immunosuppressive.

On admission, the patient's vital signs were as follows: temperature 38.5°C, blood pressure 135/78 mmHg, pulse rate 96 beats/min, respiratory rate 22 breaths/min, and oxygen saturation 89% on room air. She was alert and oriented. Physical examination revealed bilateral coarse crackles on lung auscultation without wheezing or stridor, while cardiovascular, abdominal, and neurological examinations were unremarkable. Laboratory investigations demonstrated leukocytosis (20.3×10⁹/L, 90% neutrophils), anemia (hemoglobin 9.5 g/dL), relative lymphopenia (1.5×10⁹/L), hyponatremia (133 mmol/L), and elevated inflammatory markers (CRP (C-reactive protein) 23.9 mg/dL, procalcitonin 2.12 ng/mL). Renal function was preserved (creatinine 1.0 mg/dL), and the remaining biochemical parameters were within normal limits (Table 1).

**Table 1:** Lab results WBC: white blood cells; CRP: C-reactive protein; Hb: hemoglobin; Na: sodium

Lab test	Result	Normal range	Interpretation
WBC	20.3 K/µL	4.5-11 K/µL	Elevated: suggestive of infection/inflammation
CRP	23.9 mg/dL	<1.0 mg/dL (usually)	Markedly elevated: consistent with severe inflammation/infection
Procalcitonin	2.12 ng/mL	<0.1 ng/mL	Elevated: suggestive of bacterial infection (systemic/severe)
Hb	9.5 g/dL	12-16 g/dL (female); 13-17 g/dL (male)	Low: mild to moderate anemia
Na	133 mmol/L	135-145 mmol/L	Mild hyponatremia
Creatinine	1.0 mg/dL	0.6-1.2 mg/dL	Normal: no kidney dysfunction

**Figure 1:**
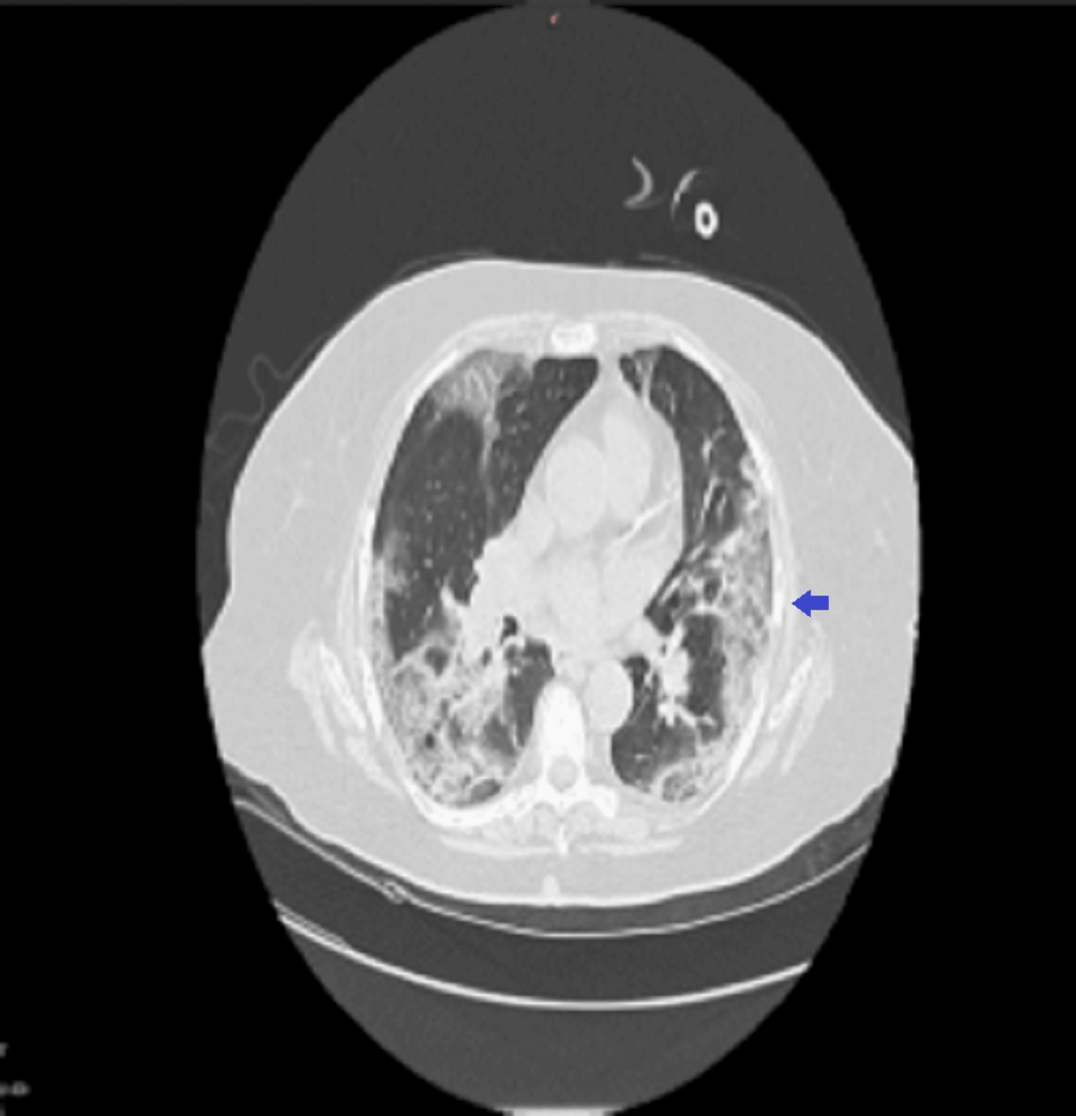
Chest CT showed extensive consolidations and ground-glass opacities in all lung lobes

The chest CT showed extensive consolidations and ground-glass opacities in all lung lobes, associated bronchiectasis, and an emphysematous cyst (Figure 1), with no evidence of pulmonary embolism. A cardiac ultrasound revealed normal ventricular dimensions, ejection fraction, and vascular status. The patient was subsequently admitted to the Pulmonology ward, where she was started on broad-spectrum antimicrobials (piperacillin-tazobactam, linezolid, moxifloxacin), antiviral therapy (remdesivir), dexamethasone, and supportive measures including proton-pump inhibitor and anticoagulation prophylaxis. Oxygen therapy was delivered via high-flow nasal cannula (HFNC) due to ongoing severe hypoxemia. Despite escalation of care, her respiratory status continued to deteriorate, and she required intubation within hours. Arterial blood gases at that point showed severe hypoxemia (partial pressure of oxygen (pO₂) 67 mmHg on fraction of inspired oxygen (FiO₂) 60%) with respiratory alkalosis. She was transferred the same day to the ICU, where sedation, neuromuscular blockade, and vasopressor support were initiated in addition to ongoing antiviral, antimicrobial, and corticosteroid treatment.

In the ICU, the patient was sedated and placed under neuromuscular blockade. She received remdesivir, intravenous (IV) dexamethasone 6 mg daily for 10 days, and continuation of the broad-spectrum antibiotics. Norepinephrine infusion was initiated at 15 µg/min to maintain a mean arterial pressure ≥65 mmHg. Over the following days, her clinical status gradually stabilized: by day 2, temperature normalized (36.2°C), oxygenation improved under controlled mechanical ventilation with FiO₂ 60% and positive end-expiratory pressure (PEEP) 12-15 cmH₂O, and hemodynamics were supported with norepinephrine titrated down to 10 µg/min. Laboratory findings showed a decrease in leukocytosis (from 45.4×10⁹/L to 31.9×10⁹/L) and improvement in CRP (from 239 mg/L to 133 mg/L), while renal function remained stable. Neuromuscular blockade was withdrawn after 48 hours, with sedation maintained. By days 3-5, FiO₂ requirements decreased to 45% with PEEP 10 cmH₂O, norepinephrine was tapered, and chest imaging showed partial resolution of infiltrates.

Despite these improvements, the patient remained intubated for 27 days. During attempts at weaning, she developed profound generalized weakness, with Medical Research Council (MRC) muscle power grading of 2/5 in all extremities, consistent with critical illness polyneuromyopathy. This was considered neuromuscular rather than purely muscular weakness, given the diffuse limb involvement, failure to generate spontaneous tidal volumes despite minimal sedation, and exclusion of focal neurological or metabolic causes. Supportive management included physiotherapy, nutritional optimization, and avoidance of unnecessary sedation, but the weakness persisted and prevented extubation.

On ICU day 28, a tracheostomy was performed to facilitate prolonged mechanical ventilation. The procedure was technically successful, and initial ventilator parameters were stable (volume control mode, FiO₂ 50%, PEEP 10 cmH₂O, partial pressure of oxygen in arterial blood (PaO₂) 78 mmHg, partial pressure of carbon dioxide in arterial blood (PaCO₂) 42 mmHg). Within hours, however, the patient developed progressive respiratory difficulty with tachypnea and increasing ventilatory pressures. Adequate ventilation was defined as maintaining PaCO₂ <45 mmHg and PaO₂ >70 mmHg on FiO₂ ≤50% with stable settings; these criteria were no longer achieved as PaO₂ fell to 60 mmHg on FiO₂ 60% and PaCO₂ rose to 52 mmHg. Physical examination revealed diffuse coarse crackles bilaterally, without stridor or pathology at the tracheostomy site. Chest imaging demonstrated worsening bilateral infiltrates without pneumothorax or cannula malposition.

**Figure 2:**
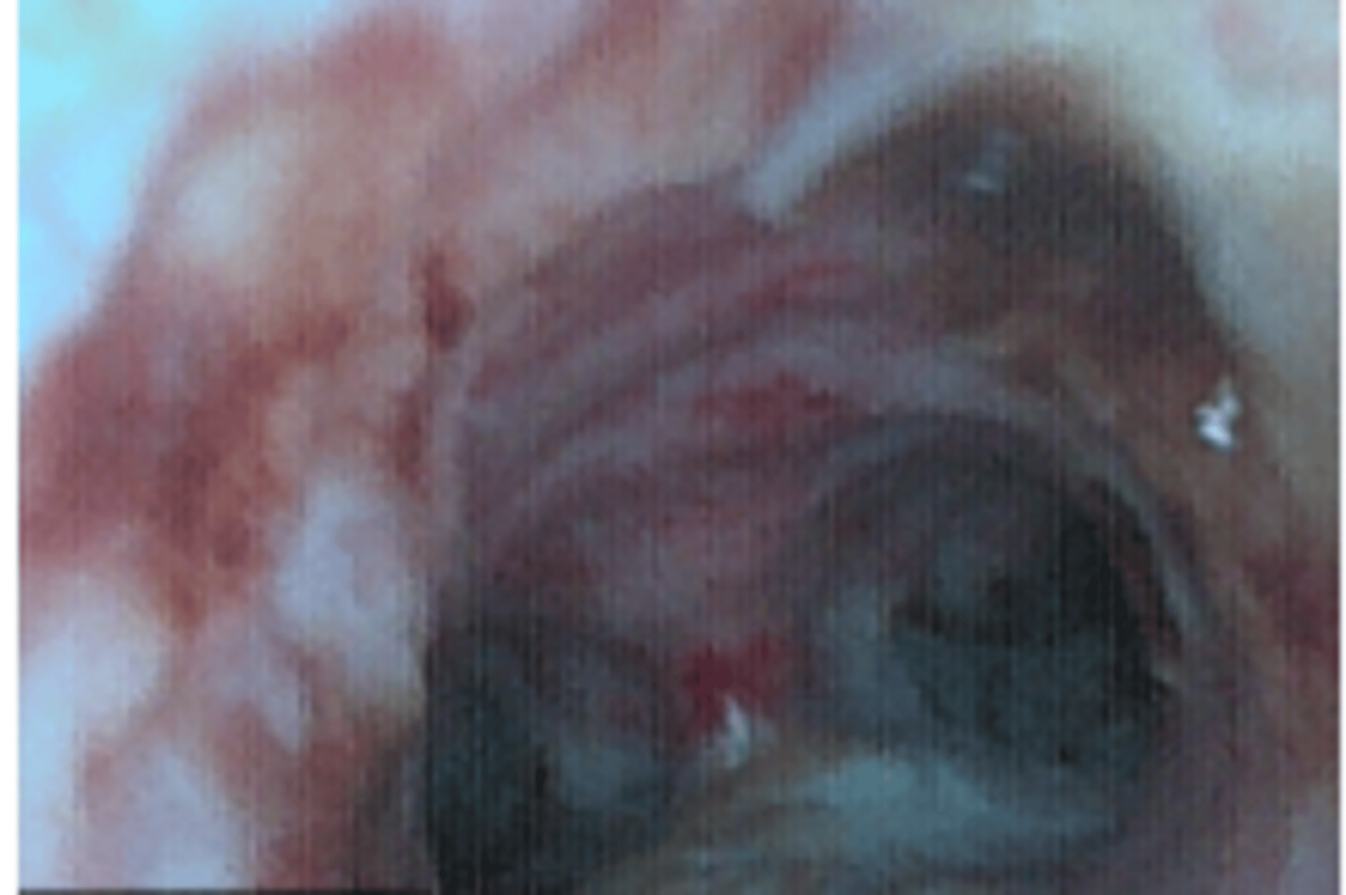
Bronchoscopy: extensive necrosis and abnormal tissue formation, which caused significant narrowing of the tracheal lumen, primarily affecting the middle and upper trachea

An urgent bronchoscopy revealed extensive necrosis and obstructive fungal plaques causing severe narrowing of the tracheal lumen, primarily involving the middle and upper trachea (Figure 2). The stenosis was graded as Cotton-Myer grade III (≈90% obstruction). Bronchoscopic debridement was performed using rigid mechanical debulking and cryoprobe extraction, successfully restoring airway patency; airway stenting was not deemed necessary. Tissue samples were collected for microbiological culture and histopathology. Cultures on Sabouraud dextrose agar, confirmed by matrix-assisted laser desorption ionization-time of flight (MALDI-TOF) mass spectrometry, identified *C. auris*. Histopathological examination demonstrated the fungal colonization of necrotic mucosal tissue without angioinvasion, consistent with invasive fungal tracheobronchitis. Antifungal susceptibility testing showed resistance to fluconazole but susceptibility to echinocandins. The patient was treated with IV anidulafungin (200 mg loading dose, followed by 100 mg daily), with continuation planned according to clinical and microbiological response.

The patient's respiratory status progressively deteriorated over the following days, as evidenced by rising oxygen requirements (FiO₂ increased from 50% to 70% with higher PEEP settings), worsening gas exchange on arterial blood gases, new bilateral consolidations on chest radiography, and persistently elevated inflammatory markers. A repeat bronchoscopy demonstrated partial improvement of the previously observed tracheal necrosis and stenosis, confirming that the clinical decline was not due to fungal airway obstruction. At this time, she was still receiving broad-spectrum antibiotics (piperacillin-tazobactam 4.5 g IV q6h and linezolid 600 mg IV q12h) alongside antifungal therapy (anidulafungin 200 mg IV loading dose and then 100 mg IV daily) and dexamethasone 6 mg IV daily. Cultures of bronchial secretions grew *Acinetobacter baumannii*, with susceptibility testing confirming a multidrug-resistant strain; antibiotic therapy was modified to meropenem (1 g IV q8h), colistin (3 MIU IV q8h), and amikacin (1 g IV once daily), tailored to the antibiogram. Steroids were discontinued after 10 days, while antifungal therapy was continued for a full course.

**Figure 3:**
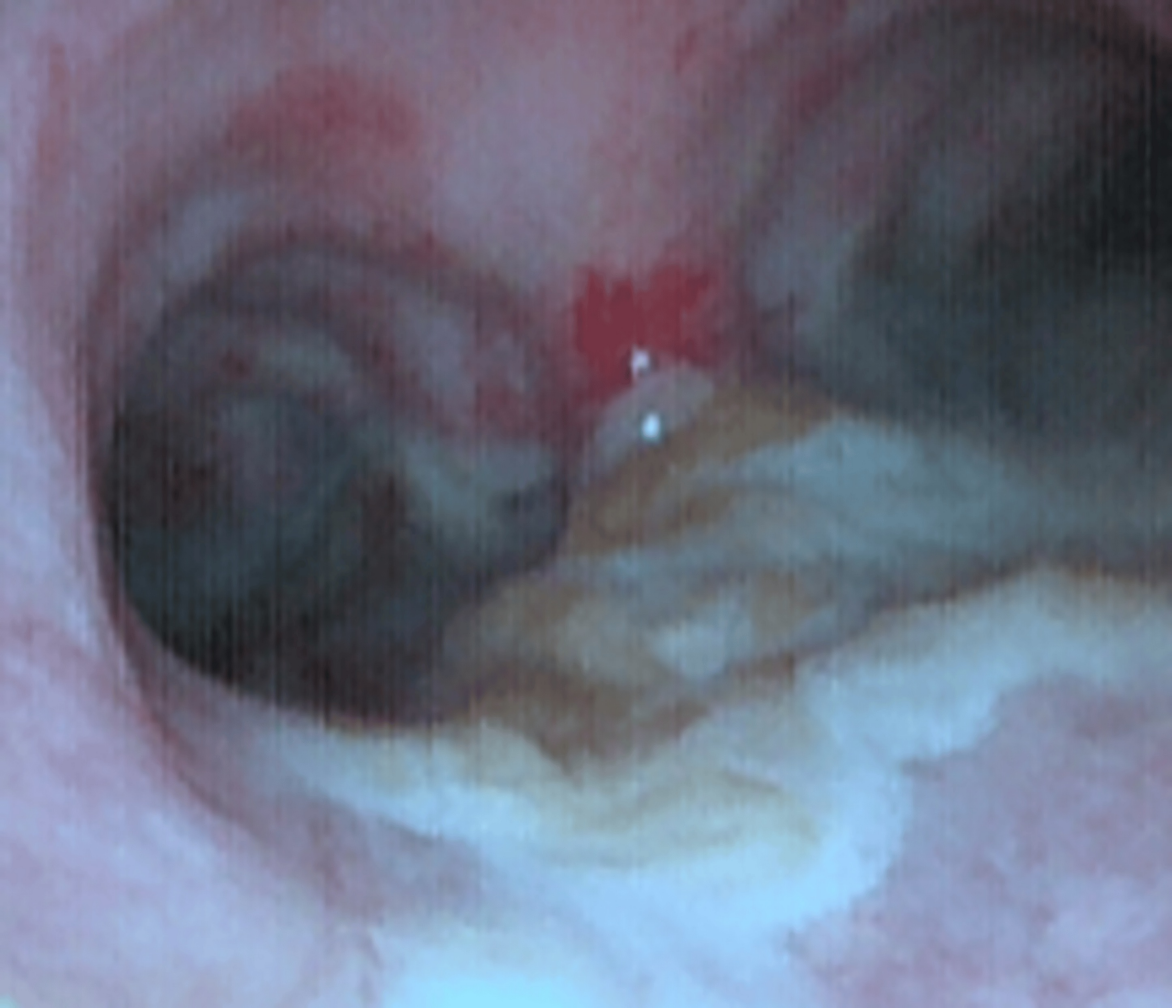
Bronchoscopy 20 days later: no residual mucosal necrosis or evidence of tracheal stenosis

A follow-up bronchoscopy performed 20 days later demonstrated the complete resolution of tracheal necrosis and stenosis (Figure 3), with a well-healed mucosa and patent lumen. At this stage, respiratory function had significantly improved: the patient was ventilated in pressure support mode with FiO₂ 35% and PEEP 8 cmH₂O, maintaining PaO₂ >80 mmHg and PaCO₂ <45 mmHg. She was able to clear secretions with suctioning assistance and tolerated spontaneous breathing trials, though generalized weakness still prevented extubation. A follow-up chest CT confirmed the regression of ground-glass opacities and consolidations with no residual airway obstruction. Fungal biomarkers (serum β-D-glucan, galactomannan) were not elevated, consistent with localized tracheobronchial infection rather than disseminated disease. Thus, both endoscopic and functional respiratory outcomes documented the resolution of the *C. auris* tracheobronchitis. The patient successfully completed antifungal treatment; however, her course was later complicated by a second episode of septic shock due to *Klebsiella pneumoniae* bacteremia, which ultimately proved fatal.

## Discussion

The World Health Organization (WHO) has designated *C. auris* as a critical priority fungal pathogen, highlighting its global public health significance [12]. A recent systematic review found that *C. auris*-associated bloodstream infections had 30-day mortality rates ranging from approximately 23% to 67% and an overall mortality between ~29% and 62% [12,13]. Resistance to fluconazole is extremely common (up to 87-100%), whereas resistance to amphotericin B ranges from 8% to 35%, and echinocandin resistance remains relatively uncommon (0-8%), though reports of emergent resistance during therapy are increasing [12,14].

Transmission in healthcare settings is facilitated by the ability of *C. auris* to persist on surfaces and equipment, survive some standard cleaning procedures, colonize the skin and other body sites without causing overt disease, and spread via medical devices and patient-to-patient contact, especially in ICUs [13,14]. Known risk factors include broad-spectrum antibiotic usage, indwelling devices such as catheters, prolonged ICU stays, mechanical ventilation, and immunocompromised status (including hematologic malignancies) [12,15]. During the COVID-19 pandemic, several reports have documented outbreaks or cases of *C. auris* infection or colonization in COVID-19 patients, likely due to increased device use and antibiotic exposure [14,15].

Management of *C. auris* infections is challenging. Echinocandins remain the first-line therapy in most guidelines, but amphotericin B (including liposomal formulations) may be considered in combination for cases of treatment failure or elevated MICs to echinocandins [16]. In vitro studies have shown that combining amphotericin B with an echinocandin can have synergistic or additive effects against some *C. auris* isolates [17]. However, clinical data are still limited, and outcomes are often poor in the setting of delayed diagnosis or severe underlying illness.

Bronchoscopic findings and microbiological confirmation supported the diagnosis of invasive *C. auris* tracheobronchitis in this case; however, current diagnostic tools have important limitations. MALDI-TOF mass spectrometry and polymerase chain reaction (PCR) provide rapid and accurate species identification but cannot differentiate colonization from invasive infection, which requires histopathological evidence of tissue invasion and necrosis [18]. Reliance on cultures or molecular assays alone therefore risks misclassification, particularly in the respiratory tract where colonization is common. This highlights the importance of integrating endoscopic, histological, and microbiological data when diagnosing invasive fungal airway disease.

COVID-19 itself may also have contributed to the development of invasive fungal infection in this patient. Severe SARS-CoV-2 infection is associated with profound immune dysregulation, including lymphopenia, impaired T-cell and NK-cell responses, and cytokine imbalance, along with prolonged ICU stays, mechanical ventilation, corticosteroid exposure, and broad-spectrum antibiotic use [19,20]. Each of these factors increases susceptibility to secondary opportunistic infections such as *C. auris*. The convergence of COVID-19-related immune dysfunction and the multidrug resistance of *C. auris* thus create a particularly high-risk scenario with significant diagnostic and therapeutic challenges.

Surveillance measures, including screening for colonization in high-risk units, routine susceptibility testing of isolates (including repeat isolates during therapy), strict infection prevention and control (IPC), environmental cleaning, and prudent antimicrobial stewardship, are essential to containing spread and improving outcomes [12,14]. In the present case, both bronchoscopic and functional outcomes demonstrated the resolution of tracheal necrosis and stenosis after antifungal therapy, illustrating that aggressive bronchoscopic debridement combined with appropriate systemic antifungal therapy can restore airway patency. Nevertheless, subsequent bacterial superinfections (ventilator-associated pneumonia and septic shock) ultimately determined the clinical course.

## Conclusions

This case illustrates a rare presentation of *C. auris* tracheobronchitis causing central airway obstruction in a critically ill patient. Diagnosis requires the integration of endoscopic, histopathologic, and microbiologic findings, as standard assays cannot reliably distinguish colonization from invasive infection. Multidrug resistance and frequent misidentification further complicate management, underscoring the need for prompt species-level identification and antifungal susceptibility testing. Patients with COVID-19 and other ICU risk factors remain particularly vulnerable. Early bronchoscopic intervention combined with appropriate echinocandin-based therapy can restore airway patency and improve outcomes. Future studies should better define diagnostic criteria and therapeutic strategies for invasive fungal airway disease to guide clinical practice.
